# ﻿*Hemipiliaavisoides* (Orchidaceae), a new species from Sichuan Province, China

**DOI:** 10.3897/phytokeys.213.90377

**Published:** 2022-11-07

**Authors:** Xue-Man Wang, Ying Tang, Pei-Hao Peng, Hua Peng

**Affiliations:** 1 College of Earth Sciences, Chengdu University of Technology, Chengdu 610059, Sichuan, China; 2 College of Tourism and Urban-rural Planning, Chengdu University of Technology, Chengdu 610059, Sichuan, China; 3 CAS Key Laboratory for Plant Diversity and Biogeography of East Asia, Kunming Institute of Botany, Chinese Academy of Sciences, Kunming 650201, Yunnan, China

**Keywords:** Arid valley, Minjiang River Valley, Orchidinae, taxonomy

## Abstract

A new orchid species, *Hemipiliaavisoides*, is described from Songpan County and Maoxian County, Sichuan Province, China. Morphologically, *H.avisoides* is most similar to *H.hui*, but can be distinguished by the combination of its involute middle lip lobe that is smaller than the lateral lobes, floral bracts that are 5 mm long and are always shorter than the ovary, a leaf that is appressed to the substrate and is adaxially green with white lines along 7–9 principal veins and the subterranean stem with a solitary sheath at its base. The floral morphology of *H.avisoides* is presented by utilising *in vivo* micro-CT scanning and 3D visualisation.

## ﻿Introduction

The genus *Hemipilia* Lindl. *sensu stricto* (Orchideae, Orchidaceae) comprises ca 10 species that are characterised by a protruding, tongue-like rostellum (Luo and Chen 2000; Chen et al. 2009b). Nevertheless, molecular phylogenies revealed *Hemipilias.s.* as monophyletic, but nested deeply within a strongly-supported clade that also included several species from closely-allied genera, i.e., *Amitostigmahemipilioides* (Finet) Tang & F.T.Wang, *A.thailandicum* Seidenf. & Thaithong, *Ponerorchisbrevicalcarata* (Finet) Soó, *P.limprichtii* (Finet) Soó and *Hemipiliopsispurpureopunctata* (K.Y.Lang) Y.B.Luo & S.C.Chen (Luo 1999; Bateman et al. 2003; Jin et al. 2014, 2017; Tang et al. 2015; Lai et al. 2021). This clade was named “the *Hemipilia* Clade” by Tang et al. (2015). In addition, the recently published species *Hemipiliagaleata* Y.Tang, X.X.Zhu & H.Peng and *H.yajiangensis* G.W.Hu, Jia X.Yang & Q.F.Wang, both of which do not possess a protruding rostellum, were also recovered as closely related to *Hemipilias.s.* in the *Hemipilia* Clade (Tang et al. 2016; Yang et al. 2022).

Given the fact that *Ponerorchis* Rchb.f. and *Amitostigma* Schltr. are paraphyletic to several taxa, including *Hemipilias.s.*, Jin et al. (2014) formally combined the monotypic *Hemipiliopsis* with *Hemipilia* and expanded the circumscription of *Ponerorchis*. Tang et al. (2015) formally proposed *Hemipiliasensu latissimo*, lumping ca 65 species into a single broadly circumscribed and monophyletic genus. Under the latter treatment, seven sections that correspond to the seven major clades in nuclear trees were also established and *Hemipilias.s.* and those basally divergent species were included in H.sect.Hemipilia (Tang et al. 2015, 2016; Yang et al. 2022).

When transferring *Ponerorchislimprichtii* to *Hemipiliasensu latissimo*, Tang et al. (2015) proposed a replacement name *H.occidensichuanensis* Y.Tang & H.Peng because of the existence of the name *H.limprichtii* Schltr. based on a different type. Unfortunately, the name *H.occidensichuanensis* turns out to be illegitimate. Schuiteman (2022) pointed out the problem and made a new combination and the correct name *Hemipiliahui* (Tang & F.T.Wang) Schuit. to replace *P.limprichtii* in *Hemipilia*. Coincidently, this particular species is the one most morphologically similar to the new taxon described in this study.

During the field trip in 2013 to collect *Hemipiliaphysoceras* (Schltr.) Y.Tang & H.Peng in Minjiang River Valley, Songpan County, Sichuan Province, China, one of the authors (Y. Tang) collected another orchid that morphologically fits into the category of *Hemipiliasensu latissimo.* It had been temporarily identified as Ponerorchiscf.limprichtii in the previous study by Tang et al. (2015). However, this taxon in Songpan not only differs in the morphology of the labellum and leaf but also diverges in DNA sequences, both of which suggest it is a potential new species (Tang et al. 2015). Here, we describe it in Hemipiliasect.HemipiliasensuTang et al. (2015) and present its floral morphology by using an *in vivo* micro-CT method.

## ﻿Methods

### ﻿Material collection

During our field investigation to Minjiang River Valley, Songpan, Sichuan, China in 7–9 June 2022, two populations of the new taxon with 12 flowering individuals were found. One population (ZJG) occurs at the same locality that was visited in 2013 by one of the authors (Y. Tang) and the other (JPY) is ca 11.2 km southwards in the Valley.

One living individual from the ZJG population and three from the JPY population with intact flowers were collected, each was packaged with soils and EPE pearl cotton in a plastic bottle and transported by air to the Key Laboratory of Stratigraphy and Paleontology, Ministry of Natural Resources for *in vivo* micro-CT scanning. After scanning, these individuals were pressed and conserved as dried specimens.

The leaf material of one individual from the JPY population was collected and dried with silica gel for DNA sequencing.

To compare the new taxon with morphologically similar species, one population of *Hemipiliahui* in Kangding, Sichuan, China was investigated in 18 June 2022. The population was found under shrubs at the elevation of ca 3470 m. Five blooming individuals with intact flowers were observed, which showed some variations in morphology but generally fit well with the description in Flora of China (Chen et al. 2009a). One individual of *H.hui* from this population was collected as a reference specimen. Digital images of herbarium specimens of *H.hui* at A, AMES, CDBI, IBSC, KUN, PE, SZ and WUK were examined.

All voucher specimens collected as part of this study were deposited at the Herbarium of Sichuan University (SZ).

### ﻿Morphological observations

The morphological description of the new taxon was mainly based on living materials. The length and width of leaves and the height of the inflorescence were measured on seven living, flowering plants in the field. The morphology of subterranean parts was described based on the four plants collected (see Material collection). The morphology of a single flower was described mainly based on the 3D mesh model reconstructed by micro-CT data.

### ﻿Micro-CT scanning and 3D Visualisation

X-ray Computed Tomography (CT) was completed at the Key Laboratory of Stratigraphy and Paleontology, Ministry of Natural Resources. The individual collected from the ZJG population (see Material collection) was finally selected for scanning and was then chosen as the holotype of the new taxon. Its inflorescence with the uppermost three flowers was scanned *in vivo* in a NIKON XTH 225ST CT scanner at a resolution of 18.6 μm and X-ray of 90 kV and 70 μA.

The 3D reconstructions were performed in the software VGSTUDIO MAX 3.0 with STL files being exported. For the 3D model of inflorescence, however, only the uppermost two flowers were reconstructed due to the trade-off between resolution and computing time. Acquired 3D mesh models were visualised and processed by the software GOM INSPECT PRO in GOM SUITE 3.1.1109.0.

### ﻿Taxon sampling, DNA sequencing and phylogenetic analyses

Based on previous studies (Tang et al. 2015, 2016; Jin et al. 2017; Lin et al. 2021; Peng et al. 2022; Yang et al. 2022), a total of 66 accessions, representing 55 taxa and all seven sections of *Hemipiliasensu latissimo*, were selected to examine the phylogenetic position of the new taxon. Two species of the genus *Brachycorythis* Lindl. were chosen as outgroups. Voucher information and GenBank accession numbers are provided in Appendix 1.

Genomic DNA extraction, primer synthesis, PCR reactions and Sanger sequencing were completed by Tsingke Biotechnology Co., Ltd. (Chengdu, China). Four DNA markers, including one nuclear (nrITS) and three plastid markers (*matK*, *trnL-F* and *trnS-trnG*), were used in this study. The primer pairs for these regions were 17SE/26SE (Sun et al. 1994), 390F/1326R (Cuénoud et al. 2002), c/f (Taberlet et al. 1991) and trnS/trnG (Hamilton 1999), respectively. All regions were sequenced for both DNA strands. Contig sequences were assembled with SEQMAN 7.1.0.

Phylogenetic reconstruction was carried out using Bayesian inference (BI) and maximum likelihood (ML) analyses. Data for the plastid regions were combined, whereas the nrITS and combined plastid DNA datasets were analysed separately according to the results of Tang et al. (2015). Each region was individually aligned with MAFFT 7.313 (Katoh and Standley 2013) in PHYLOSUITE 1.2.2 (Zhang et al. 2020) using the “L-INS-I” strategy. Alignments were then manually adjusted in PHYDE 0.9971 (Müller et al. 2010) and ambiguously aligned characters in the *trnL-F* and *trnS-trnG* datasets were excluded prior to downstream analyses. ModelFinder (Kalyaanamoorthy et al. 2017) in PHYLOSUITE 1.2.2 (Zhang et al. 2020) was used to select the best-fit model for each dataset using the Bayesian information criterion (BIC) scores. Plastid regions were finally concatenated with PHYLOSUITE 1.2.2 (Zhang et al. 2020).

The best-fit models for BI are GTR+F+I+G4 (nrITS and *matK*) and GTR+F+G4 (*trnL-F* and *trnS-trnG*) and for ML analyses they are GTR+F+I+G4 (nrITS), K3Pu+F+R3 (*matK*), K3Pu+F+R2 (*trnL-F*) and K3Pu+F+G4 (*trnS-trnG*).

Partitioned BI analyses were conducted using MrBayes 3.2.7a (Ronquist et al. 2012) on XSEDE on the CIPRES Gateway (Miller et al. 2010). The Markov chain Monte Carlo (MCMC) analyses were run for 30,000,000 generations, sampling one tree every 1,000^th^ generation. Convergence of runs was accepted when the average standard deviation of split frequencies (ASDSF) fell below 0.01. The initial 25% of sampled trees were discarded as burn-in. Partitioned ML analyses were conducted with IQ-TREE 2.1.2 (Nguyen et al. 2015) on XSEDE on the CIPRES Gateway (Miller et al. 2010) for 5,000 ultrafast (Minh et al. 2013) bootstraps. For the combined plastid dataset, each region was allowed to have its own evolution rate (“-spp”). TREEGRAPH 2.15.0-887 BETA (Stover and Muller 2010) was used to visualise the resulting trees with node support values. Nodes with a Bayesian posterior probability (BPP) ≥ 0.95 and/or a maximum likelihood bootstrap support (BS_ML_) ≥ 80 were considered as strongly supported.

## ﻿Data availability

The 3D mesh model of the uppermost two flowers on an inflorescence and photos of the corresponding micro-CT-scanned individual of *Hemipiliaavisoides* are available on Zenodo via DOI: https://doi.org/10.5281/zenodo.6832154.

## ﻿Results

### ﻿Phylogenetic reconstruction

**Figure 1.**
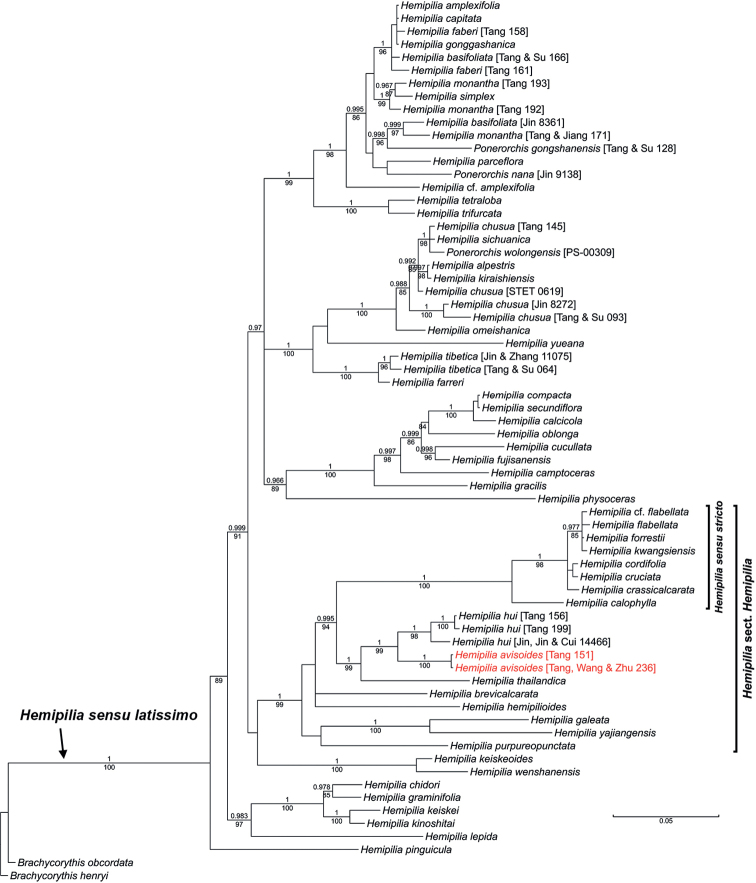
Phylogenetic placement of *Hemipiliaavisoides* sp. nov. in the Bayesian analysis of the nrITS dataset. Bayesian posterior probabilities (BPP) and maximum likelihood bootstrap supports (BS_ML_) are displayed above and below the branches, respectively. Only BPP ≥ 0.95 and BS_ML_ ≥ 80 are considered as strong supports and are shown. The scale bar denotes the estimated number of substitutions in Bayesian analysis.

**Figure 2.**
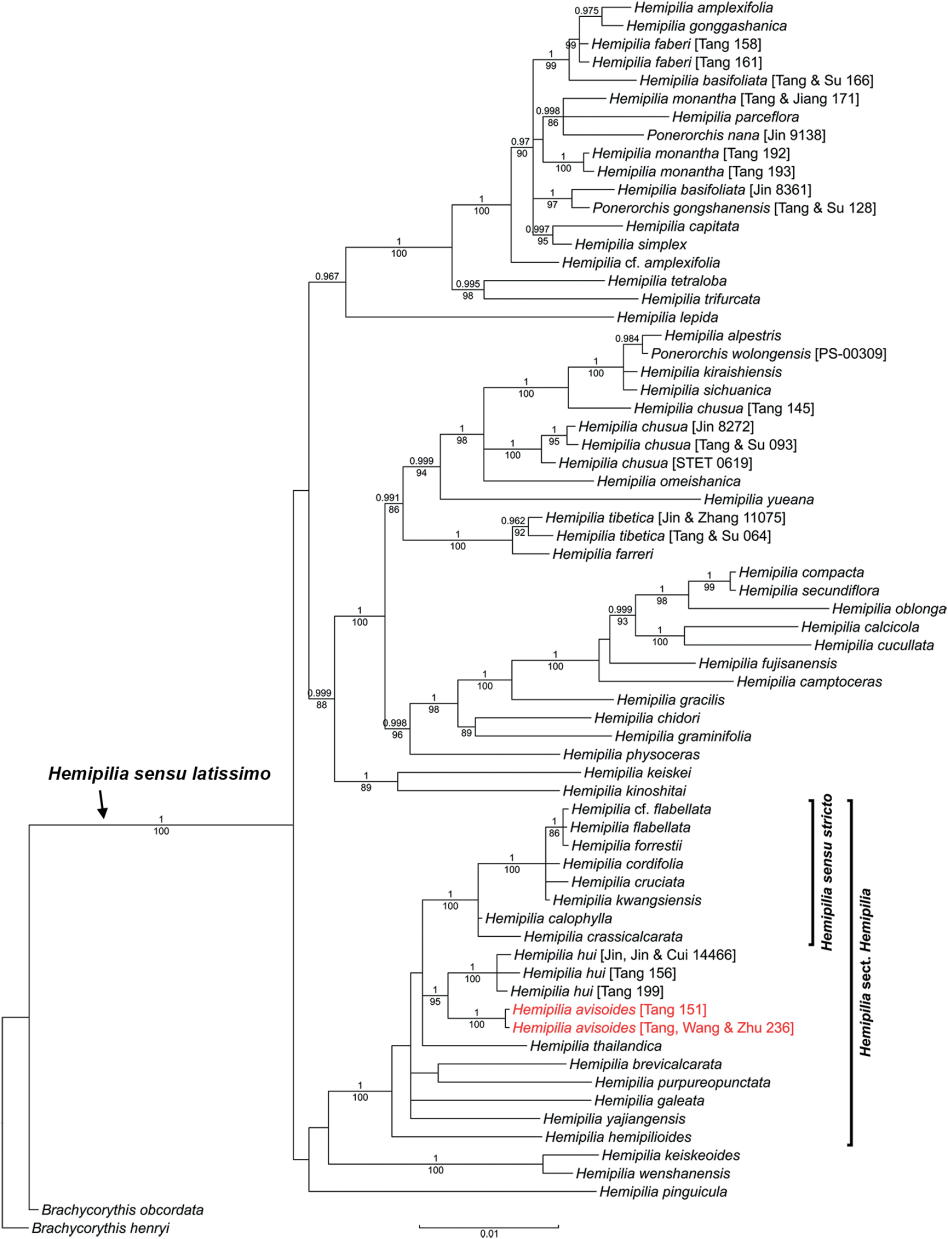
Phylogenetic placement of *Hemipiliaavisoides* sp. nov. in the Bayesian analysis of the combined plastid dataset. Bayesian posterior probabilities (BPP) and maximum likelihood bootstrap supports (BS_ML_) are displayed above and below the branches, respectively. Only BPP ≥ 0.95 and BS_ML_ ≥ 80 are considered as strong supports and are shown. The scale bar denotes the estimated number of substitutions in Bayesian analysis.

Trees reconstructed from the nrITS and combined plastid datasets in this study are similar to those of previous studies (e.g., Tang et al. 2015; Jin et al. 2017). Sequences of the accession “*Hemipiliaavisoides* [Tang, Wang & Zhu 236]” generated in this study are nearly identical to those of the accession “Ponerorchiscf.limprichtii” identified and sequenced by Tang et al. (2015). The latter accession was labelled “*Hemipiliaavisoides* [Tang 151]” in this study. The new species, represented by these two accessions, is revealed as a member of H.sect.HemipiliasensuTang et al. (2015). Both the nrITS and combined plastid trees recover the new species and *H.hui* as sister taxa with strong supports (Fig. 1: BPP = 1, BS_ML_ = 99; Fig. 2: BPP = 1, BS_ML_ = 95).

### ﻿Taxonomic treatment

#### ﻿ Hemipilia avisoides

Taxon classificationPlantaeAsparagalesOrchidaceae

Y.Tang, X.M.Wang & H.Peng sp. nov.

83473CEB-9B75-5BCC-829B-7FD1F0E18285

urn:lsid:ipni.org:names:77307801-1

**Figure 3.**
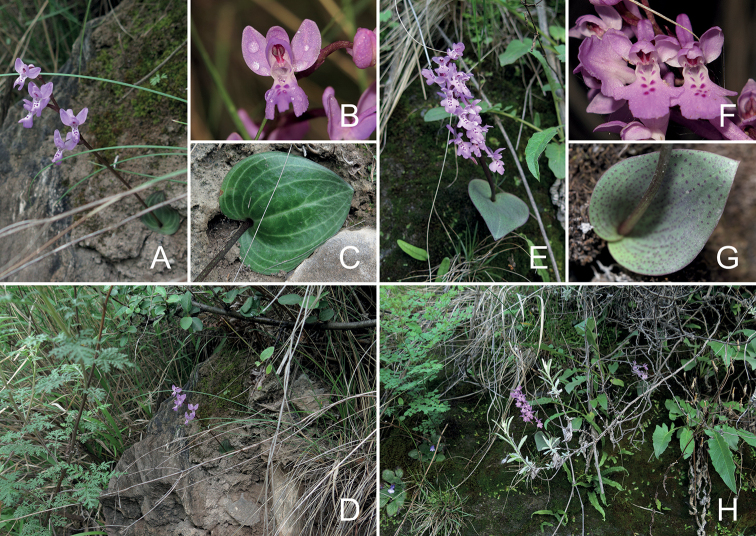
Comparisons between living plants of *Hemipiliaavisoides* sp. nov. and *H.hui* in the wild **A–D** habit, flowers, leaf and habitat of *H.avisoides***E–H** habit, flowers, leaf and habitat of *H.hui*. Photographs **A–H** by Y. Tang.

**Figure 4.**
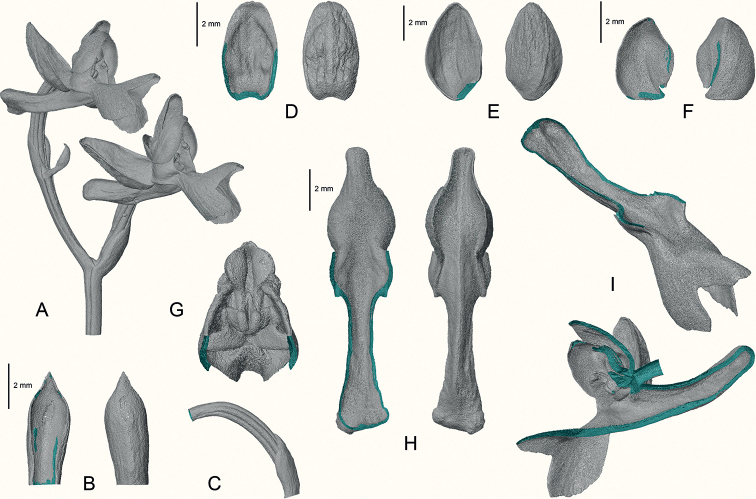
Floral morphology of *Hemipiliaavisoides* sp. nov., based on 3D mesh model reconstructed by micro-CT data **A** inflorescence with uppermost two flowers **B** ventral and dorsal views of floral bract **C** lateral view of ovary, with floral bract at base **D** ventral and dorsal views of dorsal sepal **E** ventral and dorsal views of lateral sepal **F** ventral and dorsal views of petal **G** ventral view of gynostemium **H** ventral and dorsal views of labellum **I** lateral views of labellum. The 3D model in STL format is available on Zenodo (https://doi.org/10.5281/zenodo.6832154).

**Figure 5.**
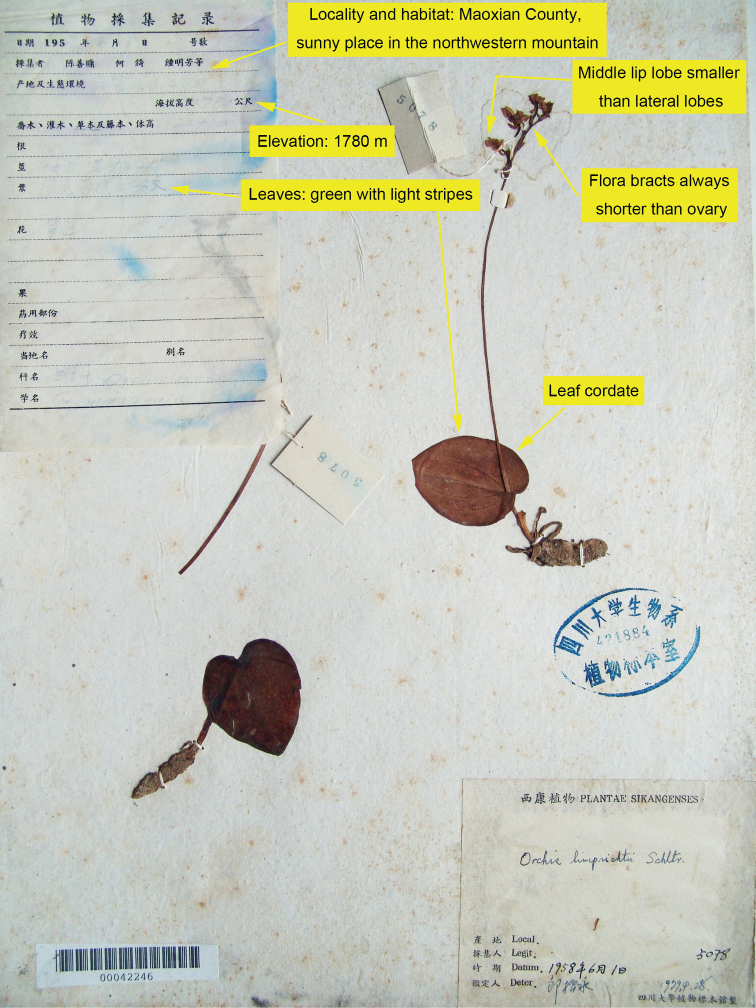
Specimen of *S.-Y. Chen, Z. He, M.-F. Zhong et al. 5078* (SZ!) identified as *Hemipiliaavisoides* in this study. Key features, which would facilitate the identification of this specimen, are highlighted and arrowed in yellow.

**Figure 6.**
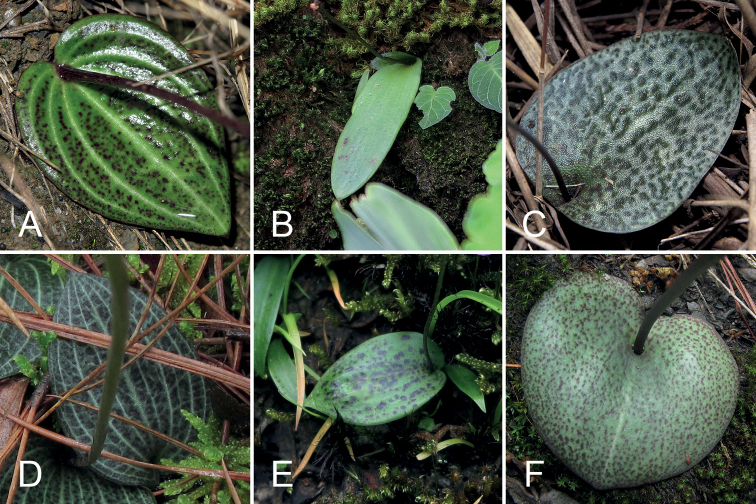
Leaves of *Hemipiliaavisoides* sp. nov. and some related species in H.sect.HemipiliasensuTang et al. (2015)**A***H.avisoides***B***H.thailandica***C***H.hemipilioides***D***H.brevicalcarata***E***H.galeata***F***H.cordifolia*. Photographs **A–F** by Y. Tang.

Figs 3A–D , 4 , 5 , 6A; see also Data availability

##### Type.

**China, Sichuan Province, Aba Tibetan and Qiang Autonomous Prefecture**, Songpan County, 9 June 2022, *Y. Tang, X.-M. Wang & Y.-T. Zhu 235* (holotype: SZ!); ibid., 9 June 2022, *Y. Tang, X.-M. Wang & Y.-T. Zhu 236* (paratype: SZ!); Maoxian County, 1 June 1958, *S.-Y. Chen, Z. He, M.-F. Zhong et al. 5078* (paratype: SZ!).

##### Diagnosis.

Similar to *Hemipiliahui* (Tang & F.T.Wang) Schuit., from which *H.avisoides* can be distinguished by a set of characteristics: mid-lobe involute, suboblong in apical view, 2.2 × 1.2 mm, trapeziform when flattened, smaller than lateral lobes; floral bracts 5 mm long, always shorter than ovary; leaf appressed to the substrate, adaxially green with white lines along 7–9 principal veins; stem subterranean, with one sheath at the base (Table 1).

**Table 1.** Comparisons in morphology between *Hemipiliaavisoides* sp. nov. and *H.hui*. Diagnostic characteristics of *H.avisoides* are in bold.

Species	*Hemipiliaavisoides*	*H.hui*
**Stem shape**	**Subterranean, with 1 sheath at the base**	Partly subterranean, with 1 or 2 (or 3) sheaths at the base
**Leaf position**	**Appressed to the substrat**e	Sub-basal
**Leaf colour adaxially**	**Green with white lines along 7–9 principal veins**, sometimes also with purple spots	Usually green with purple markings, sometimes green with white, reticulate venation or nearly uniformly green
**Flora bract shape**	Elliptic, **5 mm long, always shorter than ovary**	Lanceolate or ovate-lanceolate, lower ones nearly as long as ovary, gradually smaller upwards to shorter than ovary
**Dorsal sepal shape**	Oblong, apex rounded, sometimes concave at each side of central vein below middle	Suboblong, apex subacute
**Lateral lip lobe shape**	Pendulous, rhombic	Usually horizontal, auricular or transversely suboblong
**Middle lip lobe shape**	**Involute, suboblong in apical view, 2.2 × 1.2 mm, trapeziform when flattened, smaller than lateral lobes**, apex rounded or sometimes apiculate	Usually open and flat, subsquare, 4–5 × 3–4 mm, larger than lateral lobes, apex obtuse-rounded, sometimes slightly emarginate or shortly apiculate

##### Description.

Terrestrial, erect herbs, 8.5–31 cm tall. Tubers oblong, 2.5 cm long, 0.8 cm in diameter, neck with few roots. Stem subterranean, 2.7–5 cm long, 0.2 cm in diameter, with one sheath at the base. Sheath tubular, membranous, 1–2 cm long, pale yellow. Leaf appressed to the substrate, solitary, cordate, ovate or elliptic, 3–6.5 × 2–5.5 cm, apex acute, slightly fleshy, glabrous, abaxially purple, adaxially green with white lines along 7–9 principal veins, sometimes also with purple spots. Inflorescence terminal, erect, 3–14 cm long, 1–21-flowered, glabrous, dark purple. Flowers not secund, plum or purple plum, fragrant; floral bracts connivent to ovary, elliptic, 5 × 2.6 mm, shorter than ovary, apex acuminate, glabrous, dark purple; ovary curved, cylindrical, 10.5 mm long including pedicel, 1 mm in diameter, glabrous, dark purple. Dorsal sepal erect, oblong, cymbiform, 4.5 × 2.6 mm, apex rounded, sometimes concave at each side of central vein below middle, glabrous; lateral sepals spreading, obliquely ovate, cymbiform, 5.6 × 3.6 mm, apex obtuse, glabrous. Petals erect, connivent with dorsal sepal and forming a hood, apex bending similar to holding a fist in the other hand, obliquely ovate, 4 × 2.8 mm, apex obtuse, glabrous. Labellum spreading, broadly ovate when flattened, 7.1 × 5.4 mm, 3-lobed below middle, spurred, base collar-like raised on each side of spur entrance, glabrous, tinged white at base, disc dotted with purple; lateral lobes pendulous, rhombic, 3.4 × 2.5 mm, apex truncate, margin slightly undulate; mid-lobe horizontal, involute, suboblong in apical view, 2.2 × 1.2 mm, trapeziform when flattened, apex rounded or sometimes apiculate; spur horizontal, straight or curved upwards, cuneate, 9 mm long, ventrally carinate along central axis, entrance 2.5 mm wide, apex swollen, obtuse, 2.7 mm wide; anther reclined, 2.8 mm long, 2-locular, locules parallel and closely spaced, aubergine; pollinia 2, sectile, ovate, 1.2 × 0.7 mm; caudicles cuneate, 1.2 mm long; viscidia 2, closely spaced, oblong, transparent, each enclosed within a separate bursicle; bursicles formed by folding of rostellar arms, oblong, 0.6 × 0.3 mm; rostellum median lobe triangle, 0.7 mm long, lateral lobes grooved; stigma ventral, lobes 2, divergent, lamelliform, 1.2 × 0.5 mm, with hairs at base; auricles 2, each placed laterally at base of anther and behind collar of labellum base, 0.5 mm long.

##### Flowering.

Peaking in early June.

##### Distribution and habitat.

*Hemipiliaavisoides* is currently known from two localities in Songpan County, which are ca 11.2 km apart along the Minjiang River Valley and one locality in Maoxian County according to the collection by S.-Y. Chen et al. in 1958. Individuals of the new taxon occur under arid-valley shrubs and on moss-covered rocks (see Discussion).

##### Etymology.

Latin *avis*, bird, and suffix -*oides*, similar, alluding to appearance of flower arrangement simulating flying birds with flapped wings.

##### Chinese name.

雁字舌喙兰 (Chinese Pinyin: yànzì shéhuìlán).

##### Additional specimens examined.

*Hemipiliaavisoides*: **China, Sichuan Province, Aba Tibetan and Qiang Autonomous Prefecture**, Songpan County, 30 June 2013, *Y. Tang 151* (KUN!). *Hemipiliahui*: **China, Sichuan Province, Ganzi Tibetan Autonomous Prefecture**, Kangding City, 18 June 2022, *Y. Tang, X.-M. Wang, W.-Q. Yuan & Y.-T. Zhu 237* (SZ!); ibid., 17 June 2017, *Y.-L. Peng, Q. Yu & L.-L. Li THP-KD-1390* (CDBI!); ibid., 13 June 2014, *Y. Tang 199* (KUN!); ibid., 28 May 1981, *Z.-J. Zhao, J.-B. Shi & D.-G. Fan 114262* (SZ!); Luhuo County, 12 August 2005, *D. E. Boufford, J.-H. Chen, K. Fujikawa, S. L. Kelley, R. H. Ree, H. Sun, J.-P. Yue, D.-C. Zhang & Y.-H. Zhang 34770* (A!); Xiangcheng County, 15 July 2004, *D. E. Boufford, J.-H. Chen, S. L. Kelley, J. Li, R. H. Ree, H. Sun, J.-P. Yue & Y.-H. Zhang 30764* (A!); Daofu County, 10 June 1996, *J.-S. Yang 91-270* (IBSC!; PE!); Xinlong County, 28 June 1974, *Z.-S. Qin 06383* (CDBI!); ibid., 27 June 1974, *Z.-S. Yu 06409* (CDBI!); Yajiang County, 15 June 1961, *S. Jiang 05196* (KUN!). **China, Sichuan Province, Aba Tibetan and Qiang Autonomous Prefecture**, Xiaojin County, 2 July 2013, *Y. Tang 156* (KUN!); ibid., 21 May 1959, *Xiaojin Zu 0130* (SZ!); ibid., 21 May 1957, *J. Zhou 34* (IBSC!); Maerkang City, 16 May 1957, *X. Li 71047* (PE!; SZ!). **China, Gansu Province, Longnan City**, Wenxian County, 12 May 2007, *Baishuijiang Caijidui 4839* (PE!); ibid., 9 May 2007, *Baishuijiang Caijidui 4514* (PE!); Wudu District, 15 June 1959, *Z.-Y. Zhang 4390* (WUK!); ibid., 5 June 1959, *Z.-Y. Zhang 3379* (WUK!); ibid., 30 May 1959, *Z.-Y. Zhang 3180* (PE!; WUK!). **China, Gansu Province, Gannan Tibetan Autonomous Prefecture**, Zhouqu County, 27 May 1999, *Bailongjiang Exped. 1408* (PE!). **China**, sine loc., 1959, *Chuan Jing A 0130* (KUN!); sine loc., July 1907, *E. H. Wilson 1762* (the second individual from left on the sheet: AMES!).

##### Conservation status.

*Hemipiliaavisoides* seems narrowly distributed within the arid valley in the upper reaches of Minjiang River (see Discussion), with few populations and individuals being found. The habitat of *H.avisoides* could be easily disturbed by development as it is close to roads and villages. According to the IUCN Red List Categories and Criteria (IUCN Standards and Petitions Committee 2022), for *H.avisoides*, the area of occupancy (AOO) is 8 km^2^, the number of locations is one and the area, extent and/or quality of habitat are likely to decline due to disturbances. Moreover, the number of mature individuals is less than 50. Therefore, *H.avisoides* is here tentatively assigned to the IUCN category CR B2ab (Critically Endangered).

## ﻿Discussion

The new species *Hemipiliaavisoides* has oblong tubers, two erect anthers, two stigmas that are beneath the rostellum and two sectile pollinia with viscidium each enclosed within a bursicle. These characteristics fit well into the category of *Hemipiliasensu latissimo* (Tang et al. 2015). *Hemipiliaavisoides* has also a solitary, slightly fleshy leaf that is appressed to the substrate, which is quite reminiscent of *Hemipilias.s.* (Chen et al. 2009b). However, the median rostellum lobe of *H.avisoides* never protrudes between anther cells like that of *Hemipilias.s.*

Molecular phylogenies did, however, reveal a close relationship between *Hemipilias.s.* and *H.avisoides* in a clade (Figs 1, 2), namely, the *Hemipilia* Clade according to Tang et al. (2015). The sister relationship between *H.avisoides* and *H.hui* is strongly supported in both the nuclear and plastid trees, while they are most similar in morphology (see Taxonomic treatment and below). Moreover, *H.avisoides* and *H.hui* each occupies a relatively long branch in molecular trees (Figs 1, 2). It is also notable that one accession of *H.hui* (“Jin, Jin & Cui 14466”) shows obvious DNA sequence divergences from the other two accessions, though they still cluster into a monophyletic clade.

Amongst the specimens of *Hemipiliahui*, one collection by S.-Y. Chen et al. in 1958 caught our attention for it was gathered from Maoxian County at an elevation of 1780 m, this being close to the localities where we discovered *H.avisoides*. The environment of that region differs from the alpine habitat that *H.hui* usually favours. After careful examination, we believe that this specimen represents *H.avisoides* here described, although it was initially identified as *H.hui*. We highlighted in Fig. 5 the key features, which facilitated our identification of the specimen. Nevertheless, rather than on living plants, some subtle features could faintly be observed on pressed specimens of *H.avisoides* and its similar species. For example, the three-dimensional structure of flowers would collapse once pressed and the colours of leaves would fade away when drying. This might obscure the discrepancies between *H.avisoides* and *H.hui* or even other more distantly related species like *H.chusua* (D.Don) Y.Tang & H.Peng. We hope that the 3D mesh model reconstructed in this study (see Data availability) would become helpful for recognising *H.avisoides* in future research.

According to the spatial delimitation of the arid valley in the upper reaches of Minjiang River (Zheng et al. 2017), of the two *Hemipiliaavisoides* populations we discovered, JPY is distributed within the range of the arid valley and ZJG is closely situated next to the arid valley. The locality of the collection by S.-Y. Chen et al. in 1958 was not precisely recorded, but the elevation of 1780 m implied that the specimen was collected from the range of the arid valley in that region. However, it is notable that, as climate changes, the spatial range of the arid valley varies (Zheng et al. 2017). Focusing on vegetation type, based on our field observations, the vegetation where our collections of *H.avisoides* occur could be classified into Form. *Sophoradavidii* (Franch.) Skeels, Form. *Prunustangutica* (Batal.) Korsh. and/or Form. *Ostryopsisdavidiana* Decaisne, which are typical of arid-valley shrubs and span an elevation between ca 1700 and 2500 m (Yang 2007). To sum up, the habitats of *Hemipiliaavisoides* are mostly within the arid valley in the upper reaches of Minjiang River. To our knowledge, other orchids in the same and sympatric habitats include *Hemipiliaphysoceras*, *Habenariaacianthoides* Schltr. and *Cephalantheraerecta* Blume, although each of these species is more widely distributed overall. We believe the ecological characteristics of these orchids, including *Hemipiliaavisoides*, are worthy of future study.

The morphologically similar species *Hemipiliahui* is also distributed in Gansu Province, which is north of Sichuan Province. According to the vegetation regionalisation of China (Zhang 2007), the information of specimens (see Additional specimens examined) and the online photos (see below), the habitats of *H.hui* in Gansu probably range from arid-valley shrubs to deciduous broadleaved forests at an elevation between 1250 and 1850 m. Besides herbarium specimens, there are some photo records of *H.hui* in Gansu on the websites of Plant Photo Bank of China, PPBC and China Field Herbarium, CFH (all in Chinese; see http://ppbc.iplant.cn/tu/5920959 [by R.-B. Zhu in Zhouqu County in 21 May 2016], http://ppbc.iplant.cn/tu/5919232 [by R.-B. Zhu in Wenxian County in 16 May 2016], http://ppbc.iplant.cn/tu/5919279 [by R.-B. Zhu in Wenxian County in 16 May 2016], http://ppbc.iplant.cn/tu/7885080 [by X.-J. Liu in Chengxian County in 4 May 2021], http://ppbc.iplant.cn/tu/11011659 [by Z.-F. Bai in Wenxian County in 28 April 2021] and the remaining photos in each album and http://www.cfh.ac.cn/a5074ffa-60e3-4bf6-83a9-eb7cff45b0df.photo [by J.-H. Wang in Wenxian County in 23 April 2015]). The plants shown in those photos have a subsquare mid-lobe that is larger than the lateral lobes; therefore, we recognised them as *H.hui*. Surprisingly, their leaves are green with white, reticulate venation, which mainly resemble those of *H.brevicalcarata* Finet and *H.yajiangensis* in H.sect.Hemipilia.

A few individuals of *Hemipiliaavisoides* were observed in the field to possess conspicuously purple spots, along with white lines along 7–9 principal veins, on their leaves (Fig. 6A). However, *H.avisoides* lacks reticulate venation that is distinct in *H.brevicalcarata*, *H.yajiangensis* and the Gansu populations of *H.hui*. Occasionally, *H.hui* has a nearly uniformly green leaf as shown in another online photo (see http://hengduan.huh.harvard.edu/fieldnotes/specimens/search/specimen_detail.zpt?specimen_id=21330&full_image=skelley04179 [by S. L. Kelley in Luhuo County, Sichuan in 12 August 2005]). Despite the variation of leaf-colour patterns within each species, *H.avisoides* could be distinguished from *H.hui* as their characteristics do not overlap.

## Supplementary Material

XML Treatment for Hemipilia avisoides

## ﻿Appendix 1

**Table A1.** Voucher information and GenBank accession numbers of taxa included in phylogenetic reconstruction. Sequences generated in this study are marked with asterisks (*). Missing data are indicated with “–”.

Accession	Voucher/Reference	ITS	*matK*	*trnL-F*	*trnS–trnG*
*Brachycorythishenryi*	Jin et al. 2017	MF944262	MF945438	MF945234	–
*B.obcordata*	Jin et al. 2017	MF944263	MF945500	MF945301	–
*Hemipiliaalpestris*	Tang et al. 2015	KM651221	KM651385	KM651545	KM651627
*H.amplexifolia*	Tang et al. 2015	KM651222	KM651386	KM651546	KM651628
*H.avisoides* [Tang 151]	Tang et al. 2015	KM651296	KM651462	KM651621	KM651699
*H.avisoides* [Tang, Wang & Zhu 236]	Tang, Wang & Zhu 236	OP597820*	OP595696*	OP595697*	OP595698*
*H.basifoliata* [Jin 8361]	Jin et al. 2017	MF944399	MF945455	MF945251	–
*H.basifoliata* [Tang & Su 166]	Tang et al. 2015	KM651223	KM651387	KM651547	KM651629
*H.brevicalcarata*	Tang et al. 2015	KM651285	KM651449	KM651611	KM651689
*H.calcicola*	Tang et al. 2015	KM651279	KM651440	KM651605	KM651684
*H.calophylla*	Tang et al. 2015	KM651269	KM651433	KM651595	KM651674
*H.camptoceras*	Tang et al. 2015	KM651275	KM651439	KM651601	KM651680
*H.capitata*	Tang et al. 2015	KM651224	KM651388	KM651548	KM651630
H.cf.amplexifolia	Tang et al. 2015	KM651225	KM651415	KM651549	KM651631
H.cf.flabellata	Jin et al. 2017	KJ460050	KJ452806	MF945327	–
*H.chidori*	Tang et al. 2015	KM651287	KM651451	KM651612	KM651690
*H.chusua* [Jin 8272]	Jin et al. 2017	MF944401	MF945460	MF945257	–
*H.chusua* [STET 0619]	Jin et al. 2017	KJ460034	KJ452786	MF945189	–
*H.chusua* [Tang & Su 093]	Tang et al. 2015	KM651288	KM651452	KM651616	KM651694
*H.chusua* [Tang 145]	Tang et al. 2015	KM651290	KM651453	KM651615	KM651693
*H.compacta*	Jin et al. 2017	JN696455	KJ452796	MF945321	–
*H.cordifolia*	Jin et al. 2017	MF944329	MF945454	MF945250	–
*H.crassicalcarata*	Tang et al. 2015	KM651270	KM651434	KM651596	KM651675
*H.cruciata*	Jin et al. 2017	MF944330	MF945462	MF945259	–
*H.cucullata*	Tang et al. 2015	KM651276	KM651442	KM651604	KM651683
*H.faberi* [Tang 158]	Tang et al. 2015	KM651229	KM651391	KM651553	KM651635
*H.faberi* [Tang 161]	Tang et al. 2015	KM651230	KM651389	KM651554	KM651636
*H.farreri*	Tang et al. 2015	KM651231	KM651392	KM651555	KM651637
*H.flabellata*	Tang et al. 2015	KM651271	KM651435	KM651597	KM651676
*H.forrestii*	Jin et al. 2017	KJ460049	KJ452805	MF945326	–
*H.fujisanensis*	Tang et al. 2015	KM651280	KM651444	KM651606	KM651685
*H.galeata*	Tang et al. 2016	KT183499	KT183498	KT183500	–
*H.gonggashanica*	Tang et al. 2015	KM651233	KM651394	KM651557	KM651639
*H.gracilis*	Tang et al. 2015	KM651235	KM651397	KM651559	KM651641
*H.graminifolia*	Tang et al. 2015	KM651294	KM651458	KM651619	KM651697
*H.hemipilioides*	Tang et al. 2015	KM651238	KM651400	KM651562	KM651644
*H.hui* [Jin, Jin & Cui 14466]	Jin et al. 2017	MF944398	MF945425	MF945220	–
*H.hui* [Tang 156]	Tang et al. 2015	KM651297	KM651463	KM651622	KM651700
*H.hui* [Tang 199]	Tang et al. 2015	KM651298	KM651461	KM651623	KM651701
*H.keiskei*	Tang et al. 2015	KM651239	KM651401	KM651563	–
*H.keiskeoides*	Tang et al. 2015	KM651240	KM651402	KM651564	KM651645
*H.kinoshitai*	Tang et al. 2015	KM651241	KM651403	KM651565	KM651646
*H.kiraishiensis*	Jin et al. 2017	MF944403	MF945445	MF945241	–
*H.kwangsiensis*	Tang et al. 2015	KM651272	KM651436	KM651598	KM651677
*H.lepida*	Tang et al. 2015	KM651242	KM651404	KM651566	KM651647
*H.monantha* [Tang & Jiang 171]	Tang et al. 2015	KM651243	KM651405	KM651569	KM651650
*H.monantha* [Tang 192]	Tang et al. 2015	KM651244	KM651407	KM651567	KM651648
*H.monantha* [Tang 193]	Tang et al. 2015	KM651245	KM651406	KM651568	KM651649
*H.oblonga*	Tang et al. 2015	KM651281	KM651445	KM651607	KM651686
*H.omeishanica*	Tang et al. 2015	KM651299	KM651464	KM651624	KM651702
*H.parceflora*	Jin et al. 2017	KJ460052	KJ452808	KM651571	–
*H.physoceras*	Tang et al. 2015	KM651248	KM651410	KM651573	KM651654
*H.pinguicula*	Tang et al. 2015	KM651250	KM651413	KM651576	KM651657
*H.purpureopunctata*	Jin et al. 2017	KJ460051	KJ452807	MF945328	–
*H.secundiflora*	Jin et al. 2017	MF944406	MF945458	MF945254	–
*H.sichuanica*	Jin et al. 2017	KJ460059	KJ452815	MF945334	–
*H.simplex*	Tang et al. 2015	KM651253	KM651416	KM651578	KM651659
*H.tetraloba*	Tang et al. 2015	KM651255	KM651418	KM651580	KM651661
*H.thailandica*	Tang et al. 2015	KM651256	KM651419	KM651581	KM651662
*H.tibetica* [Jin & Zhang 11075]	Jin et al. 2017	MF944412	MF945449	MF945245	–
*H.tibetica* [Tang & Su 064]	Tang et al. 2015	KM651257	KM651421	KM651582	KM651663
*H.trifurcata*	Jin et al. 2017	KJ460055	KJ452811	KM651583	–
*H.wenshanensis*	Tang et al. 2015	KM651258	KM651422	KM651584	KM651665
*H.yajiangensis*	Yang et al. 2022	OM009240	OM009241	OM009241	OM009241
*H.yueana*	Tang et al. 2015	KM651259	KM651423	KM651585	KM651666
*Ponerorchisgongshanensis* [Tang & Su 128]	Tang et al. 2015	KM651226	KM651395	KM651550	KM651632
*P.nana* [Jin 9138]	Jin et al. 2017	MF944404	MF945475	MF945273	–
*P.wolongensis* [PS-00309]	Peng et al. 2022	MZ098270	–	–	–
